# A Prospective Cohort Study of Antipsychotic Medications in Pregnancy: The First 147 Pregnancies and 100 One Year Old Babies

**DOI:** 10.1371/journal.pone.0094788

**Published:** 2014-05-02

**Authors:** Jayashri Kulkarni, Roisin Worsley, Heather Gilbert, Emorfia Gavrilidis, Tamsyn E. Van Rheenen, Wei Wang, Kay McCauley, Paul Fitzgerald

**Affiliations:** 1 Monash Alfred Psychiatry research centre, Monash University & Alfred Hospital, Melbourne, Victoria, Australia; 2 School of Nursing and Midwifery, Monash University, Clayton, Victoria Australia; Benito Menni Complejo Asistencial en Salud Mental, Spain

## Abstract

**Background:**

Many women diagnosed with varying psychiatric disorders take antipsychotic medications during pregnancy. The safety of antipsychotic medications in pregnancy is largely unknown.

**Methods:**

We established the National Register of Antipsychotic Medications in Pregnancy in 2005. Women who are pregnant and taking an antipsychotic medication are interviewed every 6 weeks during pregnancy and then followed until their babies are one year old. The baby's progress is closely followed for the first year of life.

**Findings:**

As of April 18 2012, 147 pregnancies had been followed through to completion. There were 142 live births and data is available for 100 one year old babies. 18% of babies were born preterm, with a higher dose of antipsychotic medication correlating to an increased likelihood of premature delivery; 43% of babies required special care nursery or intensive care after birth; 37% had any degree of respiratory distress and 15% of babies developed withdrawal symptoms. Congenital anomalies were seen in eight babies. Most pregnancies resulted in the birth of live, healthy babies. The use of mood stabilisers or higher doses of antipsychotics during pregnancy increased the likelihood of babies experiencing respiratory distress or admission to Special Care Nursery or Neonatal Intensive Care Units.

**Conclusion:**

There is a great need for safety and efficacy information about the use of antipsychotic medications in pregnancy. Live, healthy babies are the most common outcome following the use of antipsychotic medication in pregnancy, but clinicians should be particularly mindful of neonatal problems such as respiratory distress.

## Introduction

Mental and behavioural disorders are the largest cause of years lived with a disability world- wide [1]. Women experience higher rates of many psychiatric disorders than men and in general receive more psychotropic medications [1]–[3]. Many of these disorders begin in adolescence and young adulthood, affecting women during their reproductive life. Most women with severe mental illness will become mothers [4]–[7] and many mothers will experience mental illness [8], [9]. Yet the care of pregnant and breastfeeding mothers with mental illness remains poorly researched and under resourced, sometimes with tragic consequences [10]. Many women with psychiatric illness require psychotropic medication treatment during pregnancy and postpartum because there is a 50% or more risk of relapse of bipolar and psychotic disorders during pregnancy and the immediate postpartum period [11], [12]. The appropriate use of psychotropic medication during pregnancy substantially reduces the risk of relapse in women with bipolar disorder [11], [13]. Untreated psychosis during pregnancy and postpartum may lead to disastrous outcomes for the woman, as well as poor maternal-infant bonding with resultant lifelong functional impairment for the developing child [14]–[16].

Antipsychotic medication is increasingly used by women of all ages [2], [17] for schizophrenia, bipolar disorder and a number of off-label indications such as depression, anxiety and borderline personality disorder [17], [18]. Multiple reviews have highlighted the lack of evidence regarding antipsychotic use in pregnancy and breastfeeding [19]–[24]. Whether antipsychotics exacerbate or mitigate the increased rate of stillbirth, prematurity, and obstetric complications seen in mothers with severe mental illness is unknown [25]–[27].

There are two major classes of antipsychotic medications – the first generation (typical) antipsychotics and the second generation (atypical) antipsychotics. Typical antipsychotics such as haloperidol have a very high affinity for dopamine D2 receptors, which results in an antipsychotic effect and a high rate of extrapyramidal side effects (EPS). Atypical antipsychotics such as quetiapine, olanzapine and risperidone have a lesser affinity for the D2 receptor, with fewer EPS side effects. Antipsychotics also impact on serotonin, histamine, acetylcholine and NMDA receptors [28]–[32]. Antipsychotics readily cross the placental barrier [33] with placental passage 23.8% for quetiapine and 72.1% for olanzapine [19], [34]. The effects of antipsychotic medication on the fetus, particularly the fetal brain, are largely unknown but concerning given adverse findings associated with perinatal exposure to other psychotropic medications [35]–[37].

Ensuring quality maternal care and a good start to early life are recognised priorities in the developed and developing world [38], [39]. Improving the care of mothers with mental illness is important to achieving such goals.

## Methods

The Australian National Register of Antipsychotic Medication in Pregnancy (NRAMP) was established in 2005 as an ongoing prospective observational cohort study (Clinical trials number NCT00686946). The workings of the register have been described in detail previously [15]. Ethics approval has been obtained from 17 ethics committees Australia-wide (for example, Alfred Hospital Ethics approval number 114/04).

The Register is based at the Monash Alfred Psychiatry research centre at the Alfred Hospital in Melbourne, Australia. Women who take antipsychotic medication during pregnancy and are able to give informed consent are prospectively recruited during pregnancy or in the first 12 months after birth. Ongoing treatment is managed by the woman's usual treating team. After providing informed consent women participate in a series of interviews, conducted in person or by telephone by a trained research nurse. With consent, information is also sought from treating clinicians and medical records.

The initial baseline interview involves a comprehensive social, family, medical, psychiatric, medication and obstetric history. The Positive and Negative Syndrome Scale (PANSS), a validated measure of psychopathology, is also administered. Women are contacted every 6–8 weeks during pregnancy and specifically asked about weight, oral glucose tolerance test results, nuchal translucency scan and blood test results, and any particular problems occurring during pregnancy.

At six weeks post partum the PANSS is repeated and the Edinburgh Postnatal Depression Scale (EPDS) is also performed [40]. Details of birthing, breastfeeding, respiratory distress and neonatal withdrawal symptoms are sought. These symptoms, which make up the Neonatal Abstinence Syndrome, include, but are not restricted to, excessive high pitched crying, pronounced moro reflex, myoclonic jerks, intermittent bouts of sneezing and/or yawning, excessive drowsiness, tremors, pronounced irritability, difficulty feeding, vomiting and loose stools [41], [42]. Babies who experience two or more of these symptoms are recorded as having experienced medication withdrawal.

At 12 weeks, 6 months and 12 months post partum the mothers are asked about their own health and the babies milestones and behaviours [43]. Developmental milestones are not covered further in this article.

For this publication descriptive statistics were performed using SPSS version 20; t-tests and chi-square tests were used where appropriate. In addition, logistic regression (via Mplus 7.1) was used to examine the effects of mothers' smoking behaviour during pregnancy (0 =  none to 4 = >20 cigarettes/day), total dose of primary and secondary antipsychotic medication in risperidone equivalents at 12 weeks antenatal, mental health DSM-IV diagnosis (psychosis vs others), antidepressant, antipsychotics(quetiaptine vs others), and mood stabiliser at 12 weeks antenatal on baby respiratory distress at delivery (no vs yes), admission to neonatal intensive care/special care nursery (no vs yes), gestation delivery week (less than 37 weeks vs over 37 weeks), and birth weight in the cohort with 1 year old babies

## Results

Data collected from 2005 up to 18 April 2012 are presented below. Over this time 192 women were recruited into the study. Of these, 49 women have been excluded from the analysis. Of the 49, 18 women were lost to follow up and the outcome of their pregnancy was unknown; 11 women were excluded as although they were mentally ill they did not require antipsychotic medication at any time during pregnancy; 20 women were excluded as they were currently pregnant and we have analysed data for completed pregnancies only. In total 147 pregnancies have been followed through to completion. Baseline characteristics of the women are shown in Table 1. These women were, for the most part, well-educated and in long-term relationships. Psychotic disorders and bipolar disorder were equally represented. Many of the women in this study had moderate to severe psychiatric disease, with most having had psychiatric hospitalisations at some time in their life. A small number of pregnancies were achieved through assisted reproductive techniques (7%), although this is approximately double the rate for the Australian general population [44]. Health related behaviours shown are poor, with smoking during pregnancy 35%, and attendance at antenatal clinics only 84%.

**Table 1:** Characteristics of mothers in pregnancy.

Baseline Characteristic	Number of women (%)
*Age, years (mean ± SD)*	32.67 (±4·7)
*Weight, kg (mean ± SD)*	75.58 (±18·77)
*BMI (mean ± SD)*	27.14 (±6·45)
*Married/de facto (n = 141)*	109 (77%)
*University degree (n = 140)*	85 (61%)
*Employed/studying during pregnancy(n = 140)*	71 (50%)
*Conception from assisted reproductive technology (n = 144)*	10 (7%)
*Diagnosis (n = 146)*	
Psychotic disorders	62 (42.5%)
Bipolar Disorder	61 (42%)
Depression	14 (10%)
Severe anxiety disorder	5 (3.5%)
Obsessive Compulsive Disorder	2 (1%)
Borderline Personality Disorder	2 (1%)
*Psychiatric admissions prior to pregnancy (n = 134)*	
At least one	108 (81%)
More than five	35 (26%)
*Smoking during pregnancy (n = 140)*	50 (35%)
*Alcohol use during pregnancy (n = 138)*	36 (26%)
*Illicit drug use during pregnancy (n = 140)*	17 (12%)
*Antenatal clinic attendance (n = 63)*	53 (84%)
*Gestational Diabetes (n = 138)*	30 (22%)

Table 2 contains data relating to the psychiatric health of the women during pregnancy. The baseline mean PANSS score was 40, which is consistent with mild psychosis. The most commonly prescribed antipsychotic agent was quetiapine followed by olanzapine. Only 11 women used typical antipsychotics. Antipsychotic doses were converted to risperidone equivalents, with the median dose at 12 weeks gestation being 3 mg/day, compared with 3.3 mg/day pre pregnancy. The rate of polypharmacy was high, with 43% of women also taking an antidepressant. The EPDS was performed at 6 weeks postpartum in 105 women. Thirty nine percent of the women tested had EPDS scores greater than 10, indicating possible depression [45].

**Table 2:** Maternal psychiatric progress and psychotropic medications.

*Total dose of antipsychotic medication in risperidone equivalents prior to pregnancy, mg (n = 142) (mean ± SD)*	3·3 (±3·5)
*Total dose of antipsychotic medication in ripseridone equivalents at 12 weeks, mg (n = 143) (mean ± SD)*	3·0 (±3·3)
*Antipsychotic used in the first trimester (n = 147)*	
Quetiapine	74 (50%)
Olanzapine	24 (16%)
Aripiprazole	19 (13%)
Risperidone	15 (10%)
Clozapine	11 (7·5%)
Haloperidol	6 (4%)
Amisulpride, Ziprasidone, Zuclopenthixol	2 each (1·5%)
Trifluoperazine	1 (0·5%)
None	5 (3·5%)
*Use of two antipsychotics (n = 144)*	16 (11%)
*Concurrent use of at least one antidepressant during pregnancy(n = 143)*	62 (43%)
*Use of a second antidepressant during pregnancy (n = 142)*	6 (4%)
*Concurrent use of a mood stabiliser during pregnancy (n = 142)*	10 (7%)
*PANSS score during pregnancy (n = 86) (mean ± SD)*	40 (±10)
*PANSS score 6 weeks post partum (n = 99) (mean ± SD)*	40·5 (±11)
EPDS score at 6 weeks post partum (n = 105) (mean ± SD)	8·4 (±7·5)
EPDS≥10	41 (39%)
*Antenatal psychiatric admission (n = 139)*	25 (18%)
*Postnatal psychiatric admission (n = 125)*	37 (30%)

Pregnancy outcomes are shown in Table 3. Overall, there were 142 live births from 147 pregnancies (including one set of twins). The rate of instrumental vaginal delivery or caesarean section was higher compared with the expected rate in Australia [44]. The reported rate of gestational diabetes was very high at 22%.

**Table 3:** Pregnancy outcomes and mode of delivery.

	Number (percent)	Expected rate in Australia [44]
*Pregnancy outcome (n = 147 pregnancies resulting in 148 babies)*		7·5 per 1000 births
Live birth	142 (95·9%)	
Miscarriage <20 weeks	4 (2·7%)	
Stillbirth ≥20 weeks	1 (0·7%)	
Ectopic pregnancy	1 (0·7%)	
*Delivery mode (n = 138)*		
Normal vaginal delivery	66 (48%)	56·8%
Instrumental vaginal delivery	17 (12%)	11·7%
Caesarean section	55 (40%)	31·5%

Of the 142 live births, 25 were born prematurely (less than 37 weeks gestation). There was a significant difference in total antipsychotic dose at 12 weeks between babies born at term and babies born prematurely (2.6 mg vs 5.0 mg p = 0.02). Using Spearman's Correlation the inverse relationship between total antipsychotic dose at 12 weeks of pregnancy and gestational age at birth remained significant, though the effect size was small (r^2^ = 0.12, p = 0.01).

Table 4 presents further data relating to the babies at birth. The mean birth weight was close to that expected, but there were a high proportion of babies with low or high birth weights. Strikingly, the occurrence of respiratory distress (ranging from a requirement for oral or nasal suctioning to full respiratory resuscitation) was 37%. Over 40% of babies required care in a Special Care Nursery (SCN) or Neonatal Intensive Care Unit (NICU), almost three times the usual rate in the Australian general population [44]. Low APGAR scores at 5 minutes following birth were also more common (APGAR scores being a global standardised measure of infant health at 1 minute and 5 minutes following birth [46]).

**Table 4:** Babies at birth.

	Number of babies (percent)	Expected rate in Australia [44]
*Female (n = 140)*	66 (47%)	48·6
*Gestational age, weeks (mean ± SD) (n = 142)*	37·6 (±5·5)	38·8
*Premature (<37 weeks) (n = 142)*	25 (18%)	8·4%
*Birth weight, kg (n = 132) mean ± SD*	3·36 (±0·7)	3·36
Less than 2.5 kg	11 (8·4%)	6·7%
More than 4.5 kg	7 (5·3)	1·8%
*Withdrawal symptoms at birth (n = 131)*	20 (15%)	
*Any degree of respiratory distress at birth (n = 129)*	48 (37%)	28%
*Admitted to special care nursery or neonatal intensive care unit (n = 131)*	56 (43%)	14·2%
*APGAR at 5 minutes <7 (n = 127)*	4 (3%)	1·5%
*Congenital anomalies (n = 130)*	8 (6%)	3·1% [59]

At birth 15% of babies displayed medication withdrawal symptoms. Babies who experienced withdrawal had been exposed to higher doses of antipsychotics at 12 weeks gestation (4.4 mg/day vs 2.7 mg/day) though this did not reach statistical significance (p = 0.162). Symptoms were prolonged in several babies, lasting 6–8 weeks.

Congenital anomalies were seen in eight babies. The anomalies reported were atrial septal defect (ASD), (in a baby with perinatal exposure to quetiapine and zuclopenthixol); cleft lip/palate and hydrocephalus in a baby (perinatal quetiapine exposure); pulmonary atresia and ASD (perinatal quetiapine exposure); abnormal renal collecting tubule and bilateral talipes in a baby (perinatal risperidone exposure), craniosynostosis, hypospadias and hypertelorism in a baby (perinatal clozapine exposure), gastroschisis and horseshoe kidney in a baby (perinatal clozapine exposure); bilateral hip dysplasia in a baby (perinatal olanzapine exposure) and CHARGE syndrome (Coloboma, Heart defects, Choanal Atresia, Retarded growth, Genital anomalies, Ear anomalies) in a baby (perinatal risperidone exposure).

At one year post-partum 96 of the babies were regarded by their mother as ‘progressing well’, whilst four babies were undergoing further assessment or treatment. Most of the mothers were also considered to be well at 12 months following the birth, being able to carry out their normal daily mothering activities, though most remained out of the paid work force.

Findings suggest that there was no statistical differences between psychosis (*n* = 39) and bipolar (*n* = 44) groups in relation to birth weight, birth weeks, respiratory distress at delivery, special care admission, and mothers' antenatal and postnatal PANSS (including positive, negative, general, and total) scores.

Results of the logistic regression suggest that the babies of mothers who used mood stabilisers were over six times (*OR* = 6.25) more likely to experience respiratory distress at delivery than babies whose mother did not use mood stabilisers during pregnancy. Moreover, higher total doses of primary and secondary antipsychotic medication in risperidone equivalents at 12 weeks antenatal increased the odds of babies being admitted to neonatal intensive care/special care nursery by 13.4% (*OR* = 1.13).

No effect was found for mothers' smoking behaviour during pregnancy, mental health DSM-IV diagnosis, antidepressant use 12 weeks antenatal and antipsychotic medication up to 12 weeks antenatal on baby respiratory distress at delivery, admission to neonatal intensive care/special care nursery, gestation delivery week, and birth weight.

## Discussion

NRAMP is the first large scale, prospective, observational study of women who take antipsychotic medication during pregnancy. While the eventual aim of the study is to identify the safest antipsychotic for use in pregnancy, these early data provide many new and valuable insights into the health and wellbeing of mothers with severe psychiatric illness and their babies. The increased rates of adverse events we describe are likely to be the result of a combination of many factors including medications, the genetic loading associated with psychiatric illness, particularly schizophrenia, increased use of alcohol, tobacco and illicit drugs, and lesser contact with antenatal care services. Despite this, by 12 months postpartum we observed good results for most mothers and babies but there are several issues that clinicians need to be cognisant of to optimize outcomes.

### Prematurity

Premature delivery has been noted in women with schizophrenia in multiple studies, though the relationship between total antipsychotic dose at 12 weeks and gestational age at delivery has not been noted previously. Whether this is a medication effect or a sign of more severe underlying illness or another comorbid factor is not clear.

### Neonatal Complications

The most unexpected finding in the babies was the very high rate of respiratory distress and admission to Special Care Nursery or Neonatal Intensive Care Units. Importantly, this may have partially resulted from the impact of mood stabiliser or antipsychotic medication use during pregnancy, as the babies of women who were taking mood stabilisers were over six times more likely than the babies of women who weren't taking mood stabilisers to experience respiratory distress at delivery. Higher doses of antipsychotic use also increased the likelihood of babies being admitted to Special Care Nursery or Neonatal Intensive Care Units.

These findings certainly highlight the potentially damaging effects of these medications on neonatal outcomes and suggest that careful consideration needs to be taken by doctors prescribing these medications during pregnancy. Interestingly, despite a heightened rate of smoking during pregnancy in women in NRAMP (35% compared to the national Australian average of 13.5% [47]), smoking did not predict neonatal complications; this is likely to reflect the relatively small numbers of women in the study. The high use of concurrent antidepressants also did not influence outcomes, despite the fact that approximately 43% of participants in NRAMP took both antipsychotics and antidepressants during pregnancy, compared to the US national antidepressant use average of 8% [48], [49]. Medication withdrawal symptoms however, might also play a significant role in neonatal complications. As we have previously reported, babies breast fed at birth experienced medication withdrawal symptoms at less than half the rate of babies who were bottle fed [50]. It has been demonstrated that haloperidol is transferred into breast milk and found in detectable quantities in breastfed infants' urine and serum [51]. Our findings suggest that other antipsychotic medications are also transmitted via breast milk at concentrations high enough to have an effect on the infant brain. The significance of this in both the short and longer term is unknown and requires further research.

### Congenital Abnormalities

Akin to other studies, we found a higher rate of congenital malformations compared with the expected rate in Australia, particularly congenital heart anomalies [52]. Of 11 babies in our cohort with perinatal clozapine exposure, two babies were born with major congenital anomalies.

### Birthweight

Earlier studies have reported higher birth weights in those exposed to atypical compared to typical antipsychotics, a relationship not examined in NRAMP due to an overwhelming preference for atypical antipsychotics [53]. The dose of antipsychotic medication in risperidone equivalents showed only a small correlation with birth weight (Spearman's rho r^2^ 0.04 p = 0.02). We found that the incidence of both large and small for gestational age birth weight was high, comparable to a previous case-control study [54].

### Maternal Metabolic Effects

NRAMP mothers experienced high rates of weight gain and gestational diabetes (GDM) during pregnancy. Increased likelihood of GDM with antipsychotic use has been reported in several other studies (OR 1.77–1.94) but to a lesser extent than in NRAMP [52], [55].

### Maternal Psychiatric Morbidity

Despite receiving medication many women were hospitalised during, or within the first year after pregnancy. The increased volume of distribution and drug clearance during pregnancy results in an increased dose being required to achieve the same serum concentrations of drug [56]. The high prevalence of EPDS scores greater than ten, supports the known high rates of postnatal depression in women with mental illness [57], with its many implications for the mother, maternal-infant bonding and child development [58].

The main limitation of this analysis is the lack of a control group to tease out the effects of medication as compared to illness. The next phase of NRAMP will address this issue. These early findings however, are still of relevance as they give clinicians a guide as to the difficulties they may encounter when treating pregnant women with mental illness that requires antipsychotic medication. There is clearly a need for further research and with ongoing and increasing recruitment we hope to determine the safest antipsychotics to use during pregnancy. It is vital to pursue this research aggressively and extend the longitudinal follow-up.

## Conclusion

In a small but growing number of women, antipsychotic medication is necessary for the mental health and well-being of the mother to be, and consequently, her baby. There is a lack of an evidence-base for clinicians and women to make informed decisions that balance the needs of the mother and baby. The NRAMP study should alert clinicians to the high risks confronting this vulnerable group. In particular, mothers face high rates of psychiatric admission and gestational diabetes, while babies frequently experience respiratory distress and medication withdrawal symptoms. Although higher antipsychotic doses and mood stabilisers may increase the risk of neonatal adverse events, clinicians and women should be reassured that positive longer-term outcomes occur in the vast majority of pregnancies. This information should be used constructively to optimise the health of mothers with mental illness and their babies.
