# Correction: A Prospective Cohort Study of Antipsychotic Medications in Pregnancy: The First 147 Pregnancies and 100 One Year Old Babies

**DOI:** 10.1371/journal.pone.0290614

**Published:** 2023-08-25

**Authors:** Jayashri Kulkarni, Roisin Worsley, Heather Gilbert, Emorfia Gavrilidis, Tamsyn E. Van Rheenen, Wei Wang, Kay McCauley, Paul Fitzgerald

There is an error in the reporting of the number of pregnancies included in this study. The original title, abstract, Table 2, Table 3, and results section of this article state that 147 pregnancies had been followed through to completion during this study. This should read 146 pregnancies instead. 2 pregnancies involved twins, leading to a total of 148 deliveries. The article’s overall results and conclusions are not affected by this reporting error. The multivariate logistic regression model included 100 babies, which is lower than the total number of deliveries reported, because subjects with missing data were excluded from multivariate logistic regression models.

The first paragraph of the results section states that 192 women were recruited into the study and that 49 women were excluded from the analysis. Among these exclusions were 20 women whose data were not included at the time of article submission, as they were still pregnant at the time the study was conducted and data analysis included completed pregnancies only. The authors clarified that the published version of this article also includes data obtained from women in this group of 20 who completed their pregnancy whilst the article was under revision, bringing the total number of pregnancies included in this study to 146.

Updated versions of Table 2 and Table 3 are provided with this notice. The total number of reported drugs in Table 2 exceeds the total number of pregnancies because some participants were prescribed more than one medication and for some participants there were changes to prescribed medication during follow-up.

**Table 2:** Maternal psychiatric progress and psychotropic medication (n = 146).

***Total dose of antipsychotic medication in risperidone equivalents*** ***prior to pregnancy*, *mg (mean ± SD) (n = 142)***		3.3 (±3.5)
***Total dose of antipsychotic medication in risperidone equivalents*** ***at 12 weeks*, *mg (mean ± SD) (n = 143)***		3.0 (±3.3)
***Antipsychotic used in the first trimester (n = 146)* ^*a*^**		
Quetiapine		74(50.7%)
Olanzapine		24(16.4%)
Aripiprazole		19(13.0%)
Risperidone		15(10.3%)
Clozapine		11(7.5%)
Haloperidol		6(4.1%)
Amisulpride		2(1.4%)
Ziprasidone		2(1.4%)
Zuclopenthixol		2(1.4%)
Trifluoperazine		1(0.7%)
None		5(3.4%)
***Use of more than two antipsychotics (n = 144)***		16(11.1%)
***Concurrent use of at least one antidepressant*** ***during pregnancy (n = 143)***		62(43.4%)
***Use of a second antidepressant during pregnancy (n = 142)***		6(4.2%)
***Concurrent use of a mood stabiliser during pregnancy (n = 142)***		10(7.0%)
***PANSS score during pregnancy (n = 86) (mean ± SD)***		40 (±10)
***PANSS score 6 weeks post partum (n = 99) (mean ± SD)***		40.5(±11)
***EPDS score at 6 weeks post partum (n = 105) (mean ± SD)***		8.4(±7.5)
EPDS ≥ 10		41(39.0%)
***Antenatal psychiatric admission (n = 139)***		25(18.0%)
***Postnatal psychiatric admission (n = 125)***		37(29.6%)

Abbreviations: SD: standard deviation; n: number of participants; PANSS: Positive and Negative Syndrome Scale; EPDS: Edinburgh Postnatal Depression Scale

^a^ participant may have more than one antipsychotic.

**Table 3:** Pregnancy outcomes and model of delivery.

	Number (in %)	Expected rate in Australia [44]
***Pregnancy outcome (146 pregnancies resulting in n = 148 babies)* ^a^**		7.5 per 1000 births
Live birth	142 (95.9%)	
Miscarriage (<20 weeks)	4 (2.7%)	
Stillbirth (≥ 20weeks)	1 (0.7%)	
Ectopic pregnancy	1 (0.7%)	
***Delivery model (n = 138)***		
Normal vaginal delivery	66(47.8%)	56.8%
Instrumental vaginal delivery	17(12.3%)	11.7%
Caesarean section	55(39.9%)	31.5%

^a^: including two sets of twins

In addition, an updated version of Table 1 that reports the total numbers assessed for each characteristic are provided with this notice.

**Table 1:** Characteristic of mothers in pregnancy (n = 146).

*Baseline Demographic*	Number of women (in %)
***Age*, *years (mean ± SD)***	32.67(±4.7)
***Weight*, *kg (mean ± SD)***	75.58(±18.8)
***BMI (mean ± SD)***	27.14(±6.5)
***Married/de facto (n = 141)***	109(77.3%)
***University degree (n = 140)***	85(60.7%)
***Employed/studying during pregnancy (n = 140)***	71(50.7%)
***Conception from assisted*** ***reproductive technology (n = 144)***	10(6.9%)
***Diagnosis (n = 146)* ^*a*^**	
Psychotic disorders	62(42.5%)
Bipolar Disorder	61(41.8%)
Depression	14(9.6%)
Severe anxiety disorder	5(3.4%)
Obsessive Compulsive Disorder	2(1.4%)
Borderline Personality Disorder	2(1.4%)
***Psychiatric admissions prior to pregnancy (n = 134)***	
At least one	108(80.6%)
More than five	35(26.1%)
***Smoking during pregnancy (n = 140)***	50(35.7%)
***Alcohol use during pregnancy (n = 138)***	36(26.1%)
***Illicit drug use during pregnancy (n = 140)***	17(12.1%)
***Antenatal clinic attendance (n = 63)***	53(84.1%)
***Gestational Diabetes (n = 138)***	30(21.7%)

Abbreviations: SD: standard deviation; n: number of participants; BMI: body mass index

^a^ participant may have more than one diagnosis.

The primary data set underlying the study cannot be shared publicly for ethical reasons; please contact the corresponding author for more information.
